# Investigation of Spatiotemporal Profiles of Single-Pulse TMS-Evoked Potentials with Active Stimulation Compared with a Novel Sham Condition

**DOI:** 10.3390/bios12100814

**Published:** 2022-10-01

**Authors:** Mayuko Takano, Masataka Wada, Reza Zomorrodi, Keita Taniguchi, Xuemei Li, Shiori Honda, Yui Tobari, Yu Mimura, Shinichiro Nakajima, Ryosuke Kitahata, Masaru Mimura, Zafiris J. Daskalakis, Daniel M. Blumberger, Yoshihiro Noda

**Affiliations:** 1 Department of Neuropsychiatry, Keio University School of Medicine, Tokyo 160-8582, Japan; 2 Teijin Pharma Limited, Tokyo 191-8512, Japan; 3 Temerty Centre for Therapeutic Brain Intervention, Centre for Addiction and Mental Health, Department of Psychiatry, Faculty of Medicine, University of Toronto, Toronto, ON M5S 1A8, Canada; 4 Shinjuku-Yoyogi Mental Lab Clinic, Tokyo 151-0051, Japan; 5 Department of Psychiatry, Faculty of Health, University of California San Diego, San Diego, CA 92161, USA

**Keywords:** transcranial magnetic stimulation (TMS), electroencephalography (EEG), sham coil, dorsolateral prefrontal cortex (DLPFC), source-based analysis

## Abstract

Identifying genuine cortical stimulation-elicited electroencephalography (EEG) is crucial for improving the validity and reliability of neurophysiology using transcranial magnetic stimulation (TMS) combined with EEG. In this study, we evaluated the spatiotemporal profiles of single-pulse TMS-elicited EEG response administered to the left dorsal prefrontal cortex (DLPFC) in 28 healthy participants, employing active and sham stimulation conditions. We hypothesized that the early component of TEP would be activated in active stimulation compared with sham stimulation. We specifically analyzed the (1) stimulus response, (2) frequency modulation, and (3) phase synchronization of TMS–EEG data at the sensor level and the source level. Compared with the sham condition, the active condition induced a significant increase in TMS-elicited EEG power in the 30–60 ms time interval in the stimulation area at the sensor level. Furthermore, in the source-based analysis, the active condition induced significant increases in TMS-elicited response in the 30–60 ms compared with the sham condition. Collectively, we found that the active condition could specifically activate the early component of TEP compared with the sham condition. Thus, the TMS–EEG method that was applied to the DLPFC could detect the genuine neurophysiological cortical responses by properly handling potential confounding factors such as indirect response noises.

## 1. Introduction

Recent technological advances in combining transcranial magnetic stimulation (TMS) and electroencephalography (EEG) have enabled direct measurement of cortical responses elicited by TMS, resulting in visualization of neural responses at the stimulation site and their propagation to other areas as time series EEG signals [1,2]. Simultaneous TMS–EEG measurements were initially applied in research at the motor cortex (M1) [3,4,5,6,7]. More recently, TMS-evoked potential (TEP) responses in non-M1 areas have garnered extensive interest and have increasingly been studied [8,9].

While the TMS–EEG method has shown great potential as a neuroscientific tool to investigate the pathophysiology of neuropsychiatric disorders [10,11], there are still concerns that have been raised about this modality. Specifically, methodological limitations raise the possibility that several potential confounding factors may have a significant impact on TEP [12,13]. In fact, since TMS stimulates not only the cerebral cortex, but also the scalp, somatosensory-evoked potentials (SSEPs), auditory-evoked potentials (AEPs) via air and bone conduction due to TMS click sounds during stimulation, and myoelectric artifacts (muscle noise) due to TMS-induced muscle contractions have been found to contaminate EEG signals, especially on the late component of TEP [14,15,16,17,18,19,20,21]. These confounding factors could be suppressed to some extent by using noise-masking methods [20,22,23,24], foam layers [22,23,24], and some analysis applications [19]. However, there is evidence that, even with these methods, it is difficult to completely eliminate peripheral stimulus-derived brain activities, especially when using suprathreshold TMS [12,15,20].

Several TMS–EEG studies thus far have been conducted using the sham condition, in which the coil position and angle were shifted from the target site on the scalp to address these confounding factors [25,26,27]. However, these methods fail to mimic somatic stimulation sensations, such as muscle contraction at the stimulation site, and may induce SSEP at another site. Recent TMS–EEG studies have also addressed the effects of auditory and somatosensory stimulation generated by TMS coils on the brain by using peripheral electrical stimulation to the scalp to mimic somatosensory stimulation, as well as by using sham coil devices that imitate the vibration and sound generated by the active coil [12,28].

Again, in the sham stimulation condition with peripheral electrical stimulation to the scalp, the magnetic field generated by an active TMS coil is not produced in principle. Thus, the elicited EEG activity generated by the conventional sham condition should be fundamentally different from TEP with active condition. In other words, the limitation of the conventional sham coil (or condition) was that the coil design (or condition) did not account for the effects of the fluctuating magnetic field generated around the coil.

To date, a few studies on M1 used sham stimulation conditions that exposed the scalp to a weak and shallow magnetic field, indicating that the sham stimulation conditions induced clearly different TEP components compared with a standard active coil stimulation [21,29]. On the other hand, for the prefrontal cortex, there have been few studies using sham stimulation coils that comprehensively mimic the active condition, accounting for the effects of the surrounding fluctuating magnetic field generated by the active coil. Therefore, to measure brain activity derived from direct stimulation to the cortex more precisely with TMS–EEG, it is necessary to separate potential artifact components from the TEP signal as much as possible, using a special sham coil that comprehensively mimics the active stimulation. To this end, the present study employed a novel special sham coil [30], which does not produce effective electromagnetism in the stimulation target area, even when sufficient suprathreshold stimulation intensity was used. Furthermore, this special sham coil magnetically stimulates only the area surrounding the target area in a manner similar to an active stimulation coil. To confirm the validity and reliability of TMS–EEG measurements, such an approach of experimental measurement and analysis is necessary.

In this study, we aimed to investigate and compare the differences between active and sham stimulation conditions with respect to the biophysical characteristics of TEP in terms of (1) amplitude (i.e., power analysis), (2) frequency (i.e., time–frequency analysis), and (3) phase (i.e., connectivity analysis including graph theory-based analysis), with a particular focus on the early and late components of TEP. Therefore, we hypothesized that TEP in the left dorsolateral prefrontal cortex (DLPFC) generated by the active coil would be more activated than the TEP elicited by the special sham coil in terms of these aspects. Given that the late component of TEP is susceptible to noise derived from peripheral stimulation [20,21], we specifically hypothesized that the early component of TEP rather than the late component would be more activated in active stimulation compared with sham stimulation.

## 2. Materials and Methods

### 2.1. Participants

A total of 28 healthy participants (13 females, mean ± standard deviation (S.D.): 33.9 ± 11.0 years old) who met the following criteria participated in the study: (1) between ages 18 and 65 at the time of obtaining consent; (2) no history of neuropsychiatric disorders, as assessed by the Mini-International Neuropsychiatric Interview; (3) normal cognitive function, as assessed by the Mini Mental State Examination (MMSE) [31] (scores of 27 or more); (4) no substance-related disorders in the 6 months prior to participation in the study; (5) no contraindications to TMS and magnetic resonance imaging such as magnetic metal implants, pacemakers, or claustrophobia; (6) no serious or unstable physical diseases; (7) no history of seizures or epilepsy; and (8) not receiving any prescriptions for central nervous system agonists, including psychotropic medications. Demographics and stimulation parameters of the participants are summarized in Table 1. The experiment was conducted in accordance with the Declaration of Helsinki and was reviewed and approved by the Ethics Committee of Keio University School of Medicine. All participants provided informed consent prior to participating in the study.

### 2.2. Experimental Procedure

Participants were asked to sit still and relax throughout the experiments, and the chair was individually adjusted to achieve the most comfortable position. Participants were instructed to open their eyes during the measurement and to stare at a fixed cross mark on the wall facing the participant. Surface electromyography was recorded from the belly of the first dorsal interosseous muscle in the right hand. First, the optimal spot for the right first dorsal interosseous muscle to evoke the largest motor-evoked potential (MEP) over the left M1 was confirmed. Next, the resting motor threshold (RMT) was determined as the minimum stimulus intensity that produced MEPs of 50 µV or more in the target muscle over 50% of the stimulus trials to the left M1 with the EEG cap in place [32].

In this TMS–EEG experiment, single-pulse TMS was applied to the left DLPFC of each participant. TMS pulses were administered with 500 ms jittering, thus the intertrial interval was set at 4.5–5.5 s. The DLPFC site (MNI coordinates: −38, 26, 44) was identified by an MRI-guided navigation system (Brainsight, Rogue Research Inc. Montréal, QC, Canada) using the individual MRI for each participant (see the Figure S1, Supplementary Materials). The stimulus intensity of the TMS was set to 120% RMT in both conditions. The same participants were subjected to the same experiment with the active coil and the sham coil on the same day and we performed block randomization so that the order of active and sham stimulations was counterbalanced across the participants.

Furthermore, participants were blinded to the stimulus conditions applied in each TMS experiment to exclude psychological effects of the participants on the EEG data during the experiment. To minimize the auditory-evoked potentials elicited by TMS click sounds, white noise was applied to participants using an earplug sound stimulation system [33]. The volume of white noise was adjusted individually to the extent that the TMS click sound was canceled out during the stimulation. The set volume was less than 90 dB for all participants. The same range of volume was used for the active and sham conditions to blind the participants to the stimulus conditions.

Finally, to ensure that the active and sham conditions were appropriately blinded for the participants, we asked each participant if they felt any differences during the TMS examination in the intensity of TMS stimulation sensations, the range of head stimulation, and the loudness of the TMS coil click sounds between each stimulation session at the end of the experiment. In this regard, we used Yes/No questions (closed questions) to avoid ambiguous answers, and if the answer was “yes” (i.e., they felt a difference between the active and sham conditions), we asked them to respond in detail about how they felt the difference.

### 2.3. TMS Conditions and EEG Recording System

In this study, a monophasic TMS stimulator (the DuoMAG MP stimulator: DEYMED Diagnostic Ltd., Hronov, Czech Republic) and a figure-of-eight butterfly coil with 2 × 70 mm diameter windings (DuoMAG 70BF; DEYMED Diagnostic Ltd., Hronov, Czech Republic) were used to conduct single-pulse TMS experiments. As for the sham condition, a special sham coil which is indistinguishable from an active stimulation coil (DuoMAG 70BFP; DEYMED Diagnostic Ltd., Hronov, Czech Republic) was used. Specifically, the sham coil used in this study generates no maximum magnetic flux in the focal region of the figure-of-eight coil. As a result, no effective current is induced in the cortical region just beneath the coil focus. However, this sham coil produces effective magnetic flux equivalent to the active stimulation in the region peripheral to the coil away from the focal area. Thus, the magnetic flux distribution outside the focal area (i.e., the induced E-field potential distribution) by sham TMS to the cortex is supposed to stimulate like the active stimulation [30]. The details of this special sham coil are described in a previously published paper [30]. All participants received 80 single-pulse TMS sessions with monophasic waveforms using active and sham coils at a same stimulus intensity of 120%RMT, with the coil positioned at 45 degrees to the midsagittal plane during TMS stimulation. The number of stimulus pulses in the study was set to 80 to increase the feasibility of the study, reduce the burden on the participants, and achieve sufficient reliability of the TEP results [34]. A TMS-compatible 64-channel EEG system and an EEG cap with silver C-ring slit electrodes (TruScan LT: DEYMED Diagnostic Ltd., Hronov, Czech Republic) were used to record the EEG during the single-pulse TMS sessions. Here, we used the TruScan EEG amplifier, which is a high-resolution TMS-compatible EEG amplifier with a sample and hold circuit system. All electrodes were referenced to an electrode connected to the right earlobe, while the ground electrode was placed on the left earlobe. EEG signals were recorded at a sampling rate of 3 kHz and the impedance between the scalp and electrodes was kept below 5 kΩ throughout the experiments.

### 2.4. EEG Preprocessing

EEG data were processed offline using the EEGLAB v2021.0 and customized scripts running on MATLAB software (R2020a, the MathWorks Inc., Natick, MA, USA) [35,36]. We used the independent component analysis (ICA) method [37,38] for noise processing and cleaning of the TMS–EEG data, following previous studies [39]. The preprocessing of EEG data is described in detail in the Supplemental Materials.

### 2.5. Global Mean Field Power and Local Mean Field Power Analysis 

Global cortical activity was assessed by averaging the global mean field power (GMFP) [40,41] of each participant across all participants, which was calculated by subtracting the mean potential of all electrodes from the potential of each electrode, dividing it by the number of electrode channels, and then taking the square root of the value. GMFP represents the standard deviation between the potentials of all electrode channels. To depict cortical activity localized to the stimulation area, we also calculated local mean field power (LMFP) [40,42,43], an index of local excitability, for the five electrodes (F3, F5, F1, F7, and AF3) in the DLPFC, using the same procedure as for the calculation of the GMFP. The GMFP and LMFP were analyzed focusing on the early component (30–60 ms) and the late component (100–200 ms), respectively, and differences between active and sham stimulation conditions were examined.

### 2.6. Time–Frequency Analyses

Custom scripts based on the FieldTrip toolbox [44] were used to conduct time–frequency analyses for frequencies from 1 to 100 Hz (three cycles at the lowest frequency and 30 cycles at the highest frequency in logarithmic intervals). Continuous Morlet wavelet transform was performed for all electrodes of all participants, and relative power was calculated as a time series before and after TMS. For the TMS-evoked total power analysis, total power was calculated by dividing the average activity of all trials at each frequency and time by the average activity of the baseline period (−300 to −100 ms) and performing a logarithmic transformation. Regarding the inter-trial phase-clustering (ITPC) analysis, the ITPC was also calculated by averaging the phase angles at each frequency and time for all trials. These time–frequency analyses were performed for both active and sham conditions, and finally the mean values of the region corresponding to the stimulated area (F3, F5, F1, F7, and AF3) were calculated for the local evaluation.

### 2.7. Connectivity and Network Analysis

To estimate the functional connectivity between brain regions for each stimulus condition at the sensor level, we used the weighted phase lag index (wPLI), which has been shown to be robust to noise in previous studies [45,46,47]. The wPLI is a value between 0 and 1, where 1 indicates the strongest functional connectivity between regions and 0 indicates no functional connectivity. The time window was set from 30 to 2000 ms, including the interval of interest, with the TMS moment set to 0, and matrices of time-series signals were created for each participant and stimulus condition. In this study, the mean functional connectivity of the wPLI was assessed using metrics averaged across stimulation sites (F3, F5, F1, F7, and AF3) for the significant frequency bands, as well as the time interval that were obtained from the time–frequency analysis. Next, we applied graph theory-based analysis to the wPLI values to evaluate the differences in the networks for each stimulus condition. The networks are represented by nodes (electrodes) and edges (connection strength between electrodes), and the main indicators of the network structure include: (1) node degree (ND), (2) clustering coefficient (CC), (3) shortest path length (PL), and (4) betweenness centrality (BC). The ND is defined as the number of edges connected to that node [48,49,50,51,52]. The method of network analysis in graph theory is described in detail in the Supplemental Materials.

### 2.8. Phase–Amplitude Coupling Analysis

The modulation index (MI) [53], which represents the phase–amplitude coupling between the two frequency bands of “phase modulation” and “amplitude modulation” was calculated using the Brainstorm toolbox [54] (See the Supplemental Materials for details on the MI calculation).

### 2.9. Source-Based Analysis

For the TEP signal source estimation, MNE software [55] was used, while the surface reconstruction was performed with FreeSurfer ver. 6.0, using a three-layer boundary element method model. Next, the surface and signal space of the boundary element method were manually registered for each participant on the EEG sensors (node, left and right anterior ear points) digitized in the Neuromag head coordinate frame. A forward model was applied to estimate the signals at the sensor level computed from the estimated neural activity at the source level [56]. Noise covariance was estimated from the individual trials using the shrink covariance method using the time window before TMS as baseline (−500 to 0 ms) [57]. Subsequently, the inverse solution was computed with a dynamic statistical parametric map [58]. Finally, the TEP and time–frequency analysis at the source level were also performed.

### 2.10. Statistical Analysis

For the GMFP and LMFP analysis, a paired t-test (two-tailed, alpha = 0.05) was applied to determine the area of the early component (30–60 ms) and late component (100–200 ms) after stimulation between both stimulus conditions [59]. The false discovery rate (FDR) was used for multiple comparison correction [60]. A cluster-based permutation test (10,000 permutations, two-tailed, alpha = 0.05) was also applied for all electrodes of each time component [59]. The total power and ITPC in the time–frequency analyses are shown as averaged values at the five electrode sites (F3, F5, F1, F7, and AF3) corresponding to the DLPFC area, and two-dimensional cluster-based permutation tests (10,000 permutations, two-tailed, alpha = 0.05, cluster alpha = 0.05) were applied to these values between the active and sham conditions, respectively. For the phase–amplitude coupling analysis, a cluster-based permutation test was applied to the computed MI values for each frequency band between the active and sham conditions by setting the ROI in the electrode sites where the graph theory-based analysis yielded significant findings. In addition, for the differences in subjective stimulus sensation between the two stimulus conditions, paired t-tests were performed, and the significance level was set at 0.05/3 (0.017) with Bonferroni correction. For comparisons between active and sham stimulation conditions at the source level on TMS-evoked responses, permutation t-tests (10,000 permutations, two-tailed, alpha = 0.05) were performed in the time intervals of 30–60 and 100–200 ms after stimulation. In addition, a two-dimensional cluster-based permutation test (10,000 permutations, two-tailed, alpha = 0.05) was performed for the time–frequency analysis of TMS-evoked responses at the source level [59,61]. A chi-square test was applied to the statistics of the results of the participant questionnaire regarding the differences in stimulation sensation between the active and sham stimulation coils.

## 3. Results

### 3.1. Global and Local Mean Field Power Analyses

**Figure 1:**
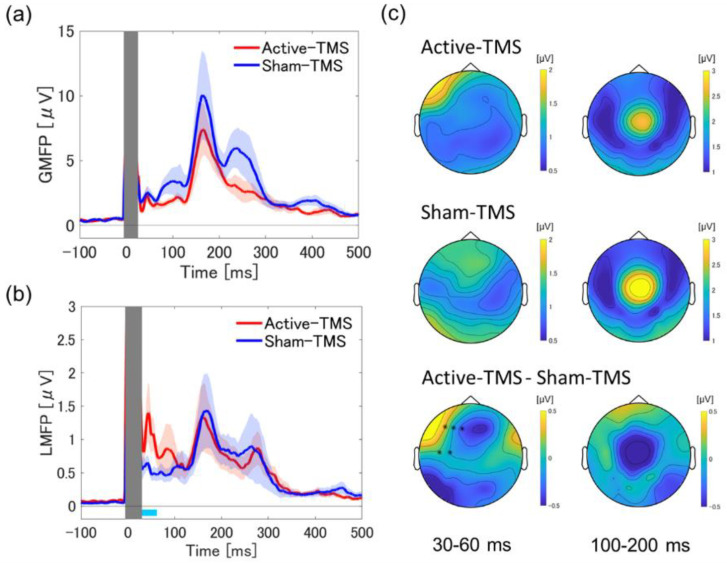
Averaged GMFP for all participants and averaged LMFP at the DLPFC stimulation site. (**a**) The upper-left panel shows the averaged GMFP waveforms (mean ± S.E.) of the TEP for all participants under active and sham conditions (active stimulation is shown as red waveform and sham stimulation is shown as blue waveform). (**b**) Likewise, the lower-left panel shows the averaged LMFP waveforms (mean ± S.E.) of the TEP for all participants confined to the DLPFC stimulation site electrodes (F3, F5, F1, F7, and AF3) (active stimulation is shown as red waveform, sham stimulation is shown as blue waveform). The light-blue bars below each graph on the left side indicate the time intervals where the statistical test showed a significant difference between the two conditions (30–60 ms). (**c**) The figure on the right shows the topoplots corresponding to the time intervals for the early component (30–60 ms) and the late component (100–200 ms) (top: active condition; middle: sham condition; bottom: active condition–sham condition). The electrode sites that showed significant differences between the two conditions were marked with a black asterisk. S.E.: standard error.

The GMFP analysis of the TEP in active and sham conditions showed no significant differences in either the early component or late component. On the other hand, in the LMFP analysis focused on the stimulated area, the LMFP in the active condition was higher than that in the sham condition in the early component (30–60 ms) (t54 = 2.18, *p* = 0.033). In contrast, no significant difference was observed for the later component of the LMFP between the two conditions (see Figure 1). Next, for the topoplots of the active and sham stimulation conditions, the following distributional differences were observed: (1) in the early component (30–60 ms), the active condition showed higher EEG power than the sham condition in the DLPFC area at the site of stimulation (*p* = 0.007); (2) No significant differences were identified in the late component (100–200 ms).

### 3.2. Graph Theory-Based Network Analyses Using wPLI Values

**Figure 2:**
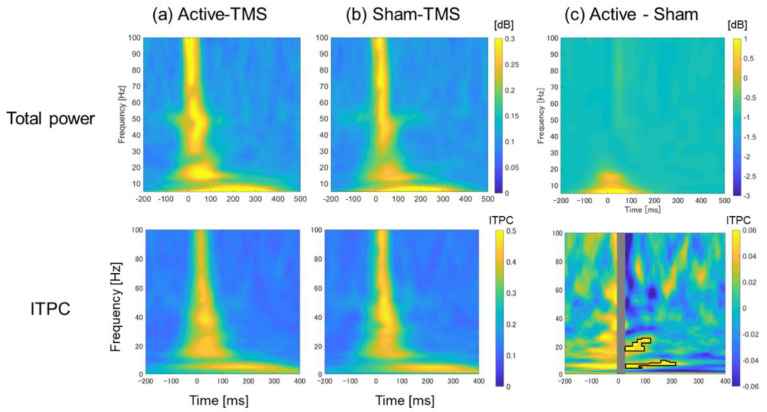
Time–frequency analyses (total power and ITPC) in the active and sham conditions. Upper panel: Total power analysis in the DLPFC stimulation electrode sites for each condition averaged over all participants. The panel (**a**) represents the total power elicited by active stimulation, panel (**b**) represents the total power elicited by sham stimulation, and panel (**c**) shows the difference in total power between the active and sham conditions (i.e., (**a**) minus (**b**)). Lower panel: ITPC analysis in the DLPFC stimulation electrode sites for each condition averaged over all participants. The panel (**a**) shows the ITPC map elicited by active stimulation, panel (**b**) shows the ITPC map elicited by sham stimulation, and panel (**c**) shows the difference in ITPC between the active and sham conditions (i.e., (**a**) minus (**b**)). The inner black line in the graph in panel (**c**) shows the area of ITPC where significant group differences were found between the active and sham conditions.

There was no significant group difference in the total power analysis for the TEP with single-pulse TMS to the DLPFC between the active and sham conditions. On the other hand, ITPC analysis for the TEP after single-pulse TMS to the DLPFC showed a significantly higher phase synchronization (p*_corrected_* = 0.032) in the active condition compared with the sham condition in θ- and β-bands in the time interval of approximately 30–200 ms post-stimulation (see Figure 2c).

### 3.3. Time–Frequency Analyses with Respect to Total Power and ITPC

**Figure 3:**
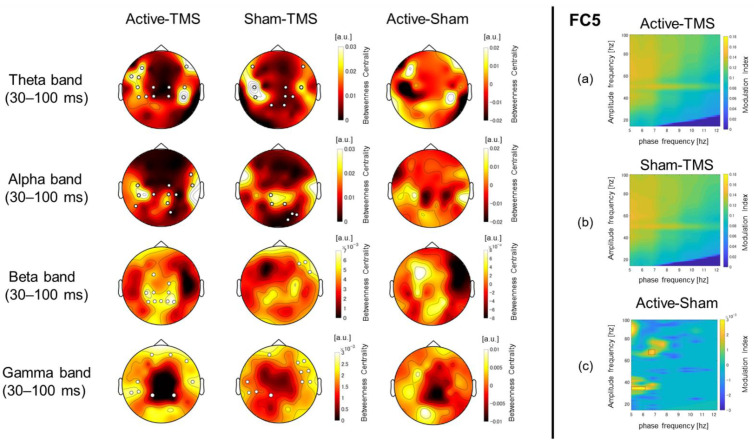
Graph theory-based network analyses for the wPLI and the comodulogram of phase–amplitude coupling expressed in the modulation index (MI). The **left** panel of Figure 3 shows the mean betweenness centrality of all participants in the active condition (**left** column), the sham condition (**middle** column), and the statistical map of differences between the active and sham conditions (**right** column). Each panel shows the results of the theta-band (4–8 Hz), alpha-band (8–13 Hz), beta-band (13–30 Hz), and gamma-band (30–100 Hz) analysis, from **top** to **bottom**, respectively. The white circles in the figure indicate the electrode sites that were regarded as hubs in the whole brain network based on the following score criteria: (1) the top 20% with the lowest value of clustering coefficients, (2) the top 20% with the shortest path lengths, (3) the top 20% with the highest degree, and (4) the top 20% with the highest value of betweenness centralities (BC). On the other side, the right panel of Figure 3 represents the comodulogram at the left fronto-central area (i.e., FC5 electrode site) that showed a significant difference in the MI values between the active and sham conditions in the interval 30–200 ms after TMS stimulation. Here, (**a**–**c**) are comodulograms showing PAC findings in the active condition, sham condition, and the difference between the two conditions, respectively.

The results of the functional connectivity analysis by the wPLI are described in detail in the Supplemental Materials (see Figure S3). Figure 3 shows the results of the BC, which is the proportion of one node that is located on the shortest path between other nodes. The differences between the active and sham conditions were barely observed in the α-band. However, the centrality in the active condition was higher at the stimulation site in the θ-band and was higher at the stimulation site and the left centro-parieto-occipital area in the β-γ bands. In the active condition, the hubs were identified in the left prefrontal and the bilateral parietotemporal areas in the θ-band, the bilateral parietotemporal areas in the α-band, the centro-parietal areas in the β-band, and the prefrontal and temporal areas in the γ-band. In the sham stimulus condition, the hubs were identified in regions similar to those in the active condition in the θ-, α-, and γ-bands. On the other hand, only in the β-band were the hubs identified in a different region (right temporal area) than in the active condition. The identified hubs are summarized in the Table S1.

### 3.4. Phase–Amplitude Cross-Frequency Coupling

For the phase–amplitude cross-frequency analyses, there were significant differences in θ-phase and γ-amplitude coupling (FC5 electrode site: t_56_ = 2.31, p*_corrected_* = 0.04) between the active and sham conditions in the DLPFC at the stimulation site, indicating that active stimulation significantly enhanced the θ–γ coupling compared with the sham condition (see Figure 3).

### 3.5. Source-Based TMS-Elicited Response and Their Time–Frequency Analyses

**Figure 4:**
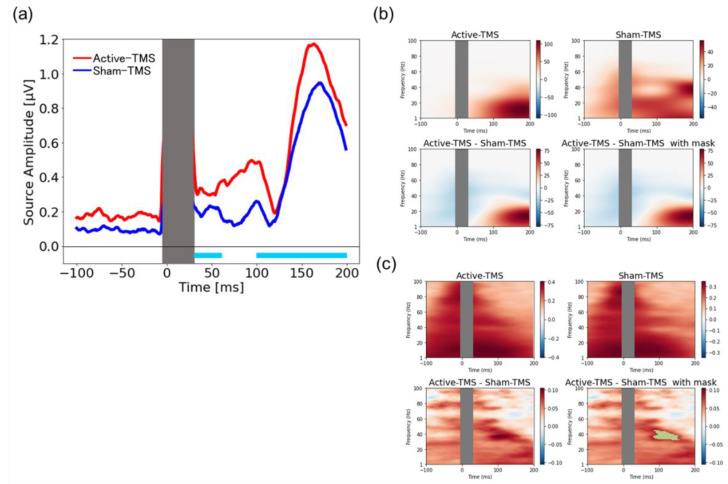
Source-based TMS-elicited response and their time–frequency analyses. (**a**) The upper panel shows the averaged TMS-elicited response for all participants confined to the source-based region, including the DLPFC stimulation site (the rostral middle frontal gyrus) with active and sham conditions (active stimulation is shown as red waveform and sham stimulation is shown as blue waveform). The intervals at which significant differences (30–60 ms) were found in permutation t-tests are indicated by the light-blue bars. (**b**) The upper panel shows the total power based on time–frequency analysis with the active condition (**upper left**) and sham condition (**upper right**) in the source-based region, including the DLPFC stimulation site. The lower-left panel shows the differences in total power between the active and sham conditions. A two-dimensional cluster-based permutation test did not detect any significant differences between the two conditions (**lower right**). (**c**) The upper panel shows the ITPC based on time–frequency analysis with active condition (**upper left**) and sham condition (**upper right**) in the source-based region, including the DLPFC stimulation site. The lower-left panel shows the differences in ITPC between the active and sham conditions. The areas showing significant differences between the two conditions in the two-dimensional cluster-based permutation test are marked with a green mask (**lower right**).

The results of the source-level signal analysis are shown in Figure 4. Permutation tests showed significant differences between the two conditions in the early component (30–60 ms) (*p* = 0.004) and the late component (100–200 ms) (*p* = 0.042) at the brain regions corresponding to the stimulated areas (see Figure 4a). The total power analysis at the source-level signal after single-pulse TMS to the DLPFC showed no significant group differences between the active and sham conditions (see Figure 4b). On the other hand, ITPC analysis at the source-level signal after single-pulse TMS to the DLPFC showed that the active condition elicited a significant increase in phase synchronization in the γ-band (around 40 Hz) around 100 ms (see green mask in the lower-right panel of Figure 4c).

### 3.6. Subjective Differences in Stimulus Sensation between Active and Sham Coils

The results of the differences in stimulus sensation (i.e., stimulus intensity, spread of the stimulus site, and loudness of the coil click sound) between active and sham stimulation coils are as follows: (1) feeling of stimulus intensity (percentage of participants who correctly answered that the sham stimulation was a sham condition: 17.9%; χ^2^(2) = 0.55, *p* = 0.46); (2) feeling of spread of the stimulus site (same as above: 14.3%; χ^2^(2) = 0.03, *p* = 0.85); and (3) feeling of loudness of the coil click sound (same as above: 7.1%; χ^2^(2) = 0.87, *p* = 0.35). Of note is that the results of the questionnaire showed no significant difference between active and sham stimulation with respect to each element of stimulus sensation.

## 4. Discussion

Using a novel sham coil, in which no effective magnetic flux is generated from the stimulus focal site, we found the following four main findings regarding the spatiotemporal profile of TEP. First, the LMFP analysis at the DLPFC stimulation site showed a significant difference between the active and sham conditions at approximately 30–60 ms. The corresponding topoplots for the time interval showed significant activation at the stimulated area in the active condition compared with the sham condition. Second, the ITPC in the time–frequency analysis showed significant differences between the two conditions in the θ- and β-bands in the time interval of 30–200 ms, indicating that the active stimulation significantly enhanced the phase synchronization in these bands. Third, the graph theory-based analysis for the wPLI showed a robust increase in BC, a hub of information processing efficiency, especially in the θ- and β-bands, around the left DLPFC stimulation site in the active condition compared with the sham condition. Fourth, the cross-frequency coupling analysis showed the significant enhancement of θ-phase and γ-amplitude coupling in the active condition compared with the sham condition around the left DLPFC stimulation site. Fifth, the source-based EEG analysis demonstrated the significant increase in TMS-elicited response in early and late components in the active condition compared with the sham condition, which reinforces the results of TMS-elicited response at the sensor level.

Previous studies noted that early components of TEP within ~80 ms after TMS are likely to reflect the stimulated cortical activity [12,26,27,62]. Furthermore, a prior TMS–EEG study that examined the paired-pulse TMS to the DLPFC also showed the TMS-evoked responses such that the test pulse (single-pulse) has a peak within 80 ms after TMS [63]. In this study, a distinct TMS-evoked response was identified in the 30–60 ms interval at the stimulation site in the active stimulation compared with the sham stimulation condition. This result, as in previous studies, may reflect more direct cortical activity at the stimulated cortical site. 

On the other hand, a previous study has shown that the TMS-evoked response around 200 ms after TMS (components closer to the late component) is significantly affected by AEP and SSEP [21]. Ross et al. quantified the TEP and SSEP elicited with the suprathreshold single-pulse TMS administered to the DLPFC in order to identify the optimal combination that minimizes the impact of SSEP [20]. In their study, three stimulation conditions were used: (1) no masking (no auditory masking, no foam, and jittered interstimulus interval (ISI)), (2) standard masking (auditory noise, foam, and jittered ISI), and (3) their own attenuated protocol (auditory noise, foam, over-the-ear protection, and unjittered ISI). Although the nature of the TMS–EEG modality makes it difficult to achieve complete sensory inhibition, their proposed attenuated protocol showed that, even with high-intensity stimulation to the DLPFC, better sensory inhibition could be implemented than with other masking methods. However, since the standard masking method was the most reliable method at the start of our study, we adopted the standard method in this study [21,64]. Note that their study also demonstrated that the standard method could reduce vertex N100-P200 complex by 22%, sound volume by 27%, and scalp sensation by 24%.

In this context, our results show no significant difference in the late component between the two conditions in the sensor-level analysis (see Figure 1). Furthermore, the source-level analysis also showed a strong significant difference between the two conditions in the early component, but only managed to reach significance in the late component (see Figure 4). Collectively, the present study demonstrates that active stimulation causes a more significant cortical activation effect on the early component of the TEP and a less significant activation effect on the late component of the TEP by using an elaborate sham stimulation coil. These results also suggest that our TMS–EEG experiments may successfully mask potential noise, including peripherally derived AEPs and SSEPs (see Figure S2). Moreover, in our study, participants reported no significant differences between active and sham stimulation conditions in the questionnaire regarding the stimulation intensity, the volume of the click sound, and the spread of the stimulation sensation. Thus, although the effects of AEP and SSEP on scalp EEG cannot be completely excluded, the late component (100–200 ms) of our TEP analysis may better reflect the evoked response to the direct stimulation of TMS in the cortex.

The sensor-based, as well as source-based, total power analyses showed no significant difference in TMS-elicited total power between the active and sham stimulation conditions. In contrast, the ITPC [65], which represents the measure of the local-phase synchrony at the site of cortical stimulation, showed that the active stimulation to the DLPFC elicited significant phase synchronization in the θ- and β-bands over the time interval of 30–200 ms in the DLPFC in the sensor-based analysis. Furthermore, the ITPC in the source-based analysis demonstrated a significant phase synchronization in the γ-band around 100 ms in the active condition compared with the sham stimulation. A previous study applied single-pulse TMS to the left DLPFC and indicated that extensive θ-band frequency modulation was elicited non-specifically to the site of stimulation within ~200 ms after TMS, while β-band frequency modulation was induced at the site of stimulation within ~100 ms after TMS [66]. In addition, a previous study applying single- and paired-pulse TMS to the left DLPFC also showed an increase in α-to-γ-band powers after the administration of single-pulse TMS [67]. Moreover, in the context of clinical research for depression, rTMS treatment to the prefrontal cortex caused increased β-band activity in the stimulated area, and the change was associated with the therapeutic mechanism of rTMS [68]. Therefore, our results, in which single-pulse TMS to the left DLPFC caused the frequency modulation in the β- and γ-bands in the stimulated regions, may reflect the shared neurophysiological mechanism with the therapeutic machinery of rTMS.

The effects of active stimulation to the left DLPFC on EEG were also evaluated by graph theory-based analysis. In particular, BC in the left fronto-parietal region in the θ- and β-bands were increased, indicating that network efficiency through this region may be enhanced by the ipsilateral stimulation. Furthermore, since more robust increases in the network efficiency in the θ- and β-bands were observed in active stimulation compared with sham stimulation, it is likely that active stimulation is causing a genuine TMS-evoked response by directly stimulating the cortex rather than spurious EEG changes. Moreover, compared with sham stimulation, active stimulation resulted in higher hubness in β-band defined by the four indexes used in the graph theory-based analysis. In addition, active stimulation significantly increased θ-phase and γ-amplitude coupling at the left fronto-central area (i.e., FC5 electrode site) with the active condition compared with the sham condition. Synchronization of oscillations with specific frequency bands is considered to represent the mechanism by which information is processed in the network [69,70]. The results of this study indicate that local active stimulation of the left DLPFC may induce more efficient information processing near the stimulation sites via functional connectivity networks.

There are some limitations in the present study. First, the time–frequency analysis in this study was restricted to the stimulation area of TMS. Therefore, future comprehensive analysis of spatiotemporal information other than the stimulation area may provide further new insights into EEG changes induced from the DLPFC stimulation using TMS–EEG. In our sham coil study, we demonstrated that the coil click sounds produced by the sham coil were 5 dB louder than those by the active coil [30]. However, this study used the same range of sound volumes in the active and sham conditions to blind the stimulus content. In addition, when our study started, the standard masking method was recommended worldwide. Note that, since the recently published study by Ross et al. showed that the ATTENUATE protocol produces more effective sensory inhibition when TMS is performed at suprathreshold intensities for the DLPFC, future TMS–EEG studies may consider employing the ATTENUATE protocol [20].

## 5. Conclusions

The present study demonstrated that the combined TMS–EEG technique could detect genuine neurophysiological cortical responses by appropriately addressing potential confounding factors that could be the sources of noise. In recent years, TMS–EEG studies have noted that it is difficult to evaluate genuinely cortex-derived TEPs because TEPs can be contaminated with mechanical and myoelectric noises from TMS, as well as the brain activities derived from peripheral stimulation (e.g., SSEP). However, our results indicate that the TMS–EEG modality can measure and evaluate the cortex-derived TEPs that are specific to the active TMS, although there are certain limitations. The application of this modality to various TMS–EEG neurophysiological paradigms accelerates the clinical application of this modality from healthy participants to patients with neuropsychiatric disorders.

## Figures and Tables

**Table 1:** Summary of demographic data and TMS parameters of the participants. RMT: resting motor threshold. MSO: maximum stimulator output; S.D.: standard deviation.

Demographic Data and TMS Parameters for This Study	
Sample size (numbers of males/females)	28 (15/13)
Age (years old)	33.9 ± 11.0 (mean ± S.D.)
RMT (%MSO)	58.2 ± 8.8

## Data Availability

TMS–EEG data and the MATLAB-based scripts used in the analyses are available upon reasonable request to the corresponding author (Y.N.).

## Supplementary Materials

The following supporting information can be downloaded at: https://www.mdpi.com/article/10.3390/bios12100814/s1, Figure S1: The coordinates of the stimulation site (MNI coordinates: x = −38, y = 26, z = 44) on the DLPFC were identified using an MRI-guided neuronavigation system (Brainsight, Rogue Research Inc, Montréal, QC, Canada) based on each participant’s individual MRI data; Figure S2: Butterfly plots for the active and sham conditions; Figure S3: EEG connectivity matrices based on wPLI values in the active and sham conditions; Table S1: The electrode sites identified as hubs by four graph theory-based indices: node degree (D), path length (PL), clustering coefficient (CC), and betweenness centrality (BC) in the active.
