# Supplementary material for: Investigation of Spatiotemporal Profiles of Single-Pulse TMS-Evoked Potentials with Active Stimulation Compared with a Novel Sham Condition
Source: Biosensors (Basel). 2022 Oct 1;12(10):814. doi: 10.3390/bios12100814 (PMC9599895; doi:10.3390/bios12100814)
Supplement: Supplementary file 1 [file biosensors-12-00814-s001.zip › biosensors-1944440-supplementary.pdf]

Supplemental Materials

Materials and Methods

EEG preprocessing and extraction of TMS-evoked EEG:

EEG data were processed offline using the EEGLAB v2021.0 and customized scripts running on the MATLAB software (R2020a, the MathWorks Inc., Natick, MA, USA) [35,36]. First, the EEG data was epoched between -2000 and 2000 ms around the TMS pulse, and the baseline correction was performed by subtracting the average signal amplitude between -500 and -150 ms. After the baseline correction, channel electrodes with high variability such as poor contact were automatically identified and removed (mean±S.D.: 3±2 channels). The threshold for electrode removal was set as the median z-score greater than 3 of all epochs in the 20–300 ms time interval after TMS of each electrode. On the other hand, for the electrodes (F5, F3, F1, F7, AF3, FC3, and FC5) corresponding to the stimulation site (DLPFC), we pre-set them not to be automatically excluded. Artifacts with large amplitude, such as TMS-evoked muscle activity, affect subsequent ICA processing and contaminate the TMS-evoked EEG until approximately 15-20 ms after TMS [13]. In addition, we applied the technique of Rogasch et al. for each participant, EEG data with amplitudes greater than 150 μV immediately after TMS were cut off to minimize the possibility of TMS-induced muscle activity noise contaminating the TEP. Note that even employing that technique, we confirmed that the interval of EEG data to be cut out is within 25 ms [19] . Next, to remove the TMS pulse artifacts on the EEG, zero padding was performed with respect to the -5 ms to 30 ms interval immediately after the TMS. Subsequently, EEG data were downsampled to 1,000 Hz, and epochs containing large noise with an amplitude of 1,000 μV or more were automatically identified and visually removed. Afterwards, a first round of independent component analysis (ICA) (EEGLAB FastICA 2.5) was performed on the preprocessed EEG data to identify and remove the TMS-evoked muscle artifact components (mean±S.D.: 8.5±7.4 ICs) on the EEG [37]. After the initial ICA process, the pop_tesa_interpdata function was applied to perform cubic interpolation between -5 ms and 30 ms, and a band-pass filter between 0.5 Hz and 100 Hz (forward-backward 4th order Butterworth filter) and a notch filter between 48 Hz and 52 Hz were also performed. The interval from -5 ms to 30 ms was zero-padded again to remove elements not related to TMS-induced activity and make it easier to identify artifact components (blink, eye movement, muscle activity, electrode noise, etc.), and a

second ICA (EEGLAB infomax) using the pop_runica function was performed [38]. In the second ICA, the remaining artifacts such as eye movement/blink, muscle noise, electrode noise, TMS-evoked muscle noise, and heartbeat noise were manually removed (19.4±7.8 ICs). In the two-step ICA process, the pop_tesa_compselect function of the TMS-EEG Signal Analyzer (TESA 1.1.1) [39], an open-source, semi-automatic ICA artifact detection algorithm embedded in the EEGLAB extension, was used to characterize each IC based on the patterns of EEG frequency, activity power spectra, amplitude, topographic distribution, and time course change. Then, the investigator visually identified whether they were noise or not. Thereafter, the zero-padded intervals (-5 ms to 30 ms) were cubically interpolated and referenced to the global average. Furthermore, missing and/or removed channels were interpolated using the spherical interpolation. Finally, preprocessed denoised and epoched EEG data (mean ± S.D.: 73.2 ± 4.2 out of 80 epochs) were averaged and TEP was extracted for each participant. The mean TEP was delineated by averaging the TEPs of all participants.

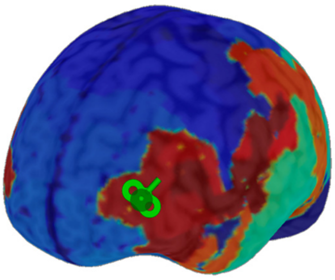

Figure S1. The coordinates of the stimulation site (MNI coordinates: x=-38, y=26, z=44) on the DLPFC were identified using an MRI-guided neuronavigation system (Brainsight, Rogue Research Inc, Montréal, QC, Canada) based on each participant's individual MRI data.

Graph theory-based analyses using wPLI values:

The clustering coefficient represents the degree to which the network is organized in local regions [48,49]. In this analysis, all local clustering coefficients were averaged to calculate the clustering coefficient of the graph, which ranges from 0 to 1. The shortest path length is the minimum number of steps required to reach from arbitrary node to any other node. The average of the inverse of the shortest path length indicates the global communication efficiency of the network, i.e., the global efficiency [50]. The higher the value of global efficiency, the higher the communication efficiency between network nodes [49]. Betweenness centrality is the rate of all shortest paths in a network that pass through given nodes. In other words, nodes with high betweenness centrality are located on the shortest path between

other nodes, thus a significant betweenness centrality represents an index of nodes that are important for connections in the graph. We also classified the brain hubs of the network based on the four node parameters: clustering coefficient, shortest path length, node degree, and betweenness centrality. The identification of significant hubs was defined as those that met the following criteria according to a previous study [49]: (1) the top 20% with lowest clustering coefficient values; (2) the top 20% with shortest path lengths; (3) the top 20% with highest degrees; and (4) the top 20% with highest betweenness centrality values. Specifically, scoring was performed based on how well these four criteria were met (hub score = 1 if the condition was satisfied), and the total hub score was calculated. Then, if the hub score ≥ 2, the node was defined as a significant hub [51,52] . Graph theory analysis was calculated by custom scripts based on the FieldTrip toolbox [44] . See below for the formulas used in the graph theory analysis.

Since the wPLI connectivity analysis conducted in this study does not include directionality, the following equation was used to calculate node degree (D) in graph theory.

$$D(i) = \sum_{j=1}^{N} k_{ij}$$

Here, k_{ij} indicates the strength of the connection between electrode *i* and *j*, defined as one if it exceeds the threshold and 0 if it is below the threshold. N is the total number of electrodes. The clustering coefficient (CC) was calculated using the following equation.

$$CC(i) = \frac{t_i}{D(i)(D(i)-1)/2}$$

Here, t_i denotes the number of triangles adjacent to each node. The shortest path length (PL) indicates the number of least edges in the connection between a node pair. The betweenness centrality (BC) was calculated based on the following formula.

$$BC(i) = \sum_{h,j \in N} \frac{sp_{hj}(i)}{sp_{hj}}$$

Here, sp_{hj} is the number of the shortest paths from node *h* to node *j*, and $sp_{hj}(i)$ is the number of paths that pass through node *i* within the shortest paths from node *h* to node *j*. BC was calculated for all nodes *i*.

Phase-amplitude coupling analysis [53]**:**

For the EEG signals from 30 to 500 ms after TMS, phase waveforms were filtered into delta, theta, alpha, and beta band waveforms, and amplitude waveforms were filtered into theta, alpha, beta, and gamma band waveforms for all electrodes. A Hilbert transform was applied for each waveform to obtain a time series of phase and amplitude envelope signals. The phase was divided into eighteen bins, and the amplitude time series signals corresponding to those bins were averaged. To quantify the phase-amplitude coupling, the average amplitude in each phase bin was divided by the sum of all amplitudes across bins to calculate the relative amplitude distribution for each participant.

The mean amplitude of each amplitude corresponding to each phase bin in the EEG time series data was normalized and calculated by the following equation.

$$P(j) = \bar{a} / \sum_{k=1}^{N} \overline{a_k}$$

Here, *a* is the mean amplitude in one bin and *N* is the total number of phase bins. In information theory, the entropy of a random variable is defined to be the average level of information inherent in the possible outcomes of that random variable, which is calculated from the series of events and the probability distribution of each event by the following formula of Shannon entropy

$$H(P) = -\sum_{j=1}^{N} P(j) \log P(j)$$

P denotes the vector of normalized mean amplitudes for each phase bin. Furthermore, the Kullback-Leibler (KL) distance, a measure to quantify the difference between the two probability distributions, was calculated.

$$KL(P,U) = \log N - H(P)$$

U is the uniform distribution and *P* is the probability distribution of the amplitude obtained by analyzing the experimental data. Finally, the MI value was calculated using the following equation. If the mean amplitude corresponding to each phase bin is uniformly distributed, then *P* is equal to *U*, resulting in the MI of the phase-amplitude coupling index is zero.

$$MI = KL(P,U)/\log N$$

Results

TMS-evoked potentials (TEPs) between the active and sham conditions:

Single-pulse TMS with 120%RMT stimulus intensity applied to the left DLPFC produced the characteristic butterfly plots for both active and sham conditions (**Figure S2**). The figure showing the difference in the topoplots after stimulation (i.e., approximately 30–60 ms post-stimulus) between the active and sham conditions showed that TMS-evoked potentials were higher for the active stimulation at the DLPFC stimulation site, while TMS-evoked potentials were rather higher for the sham stimulation at the surrounding area.

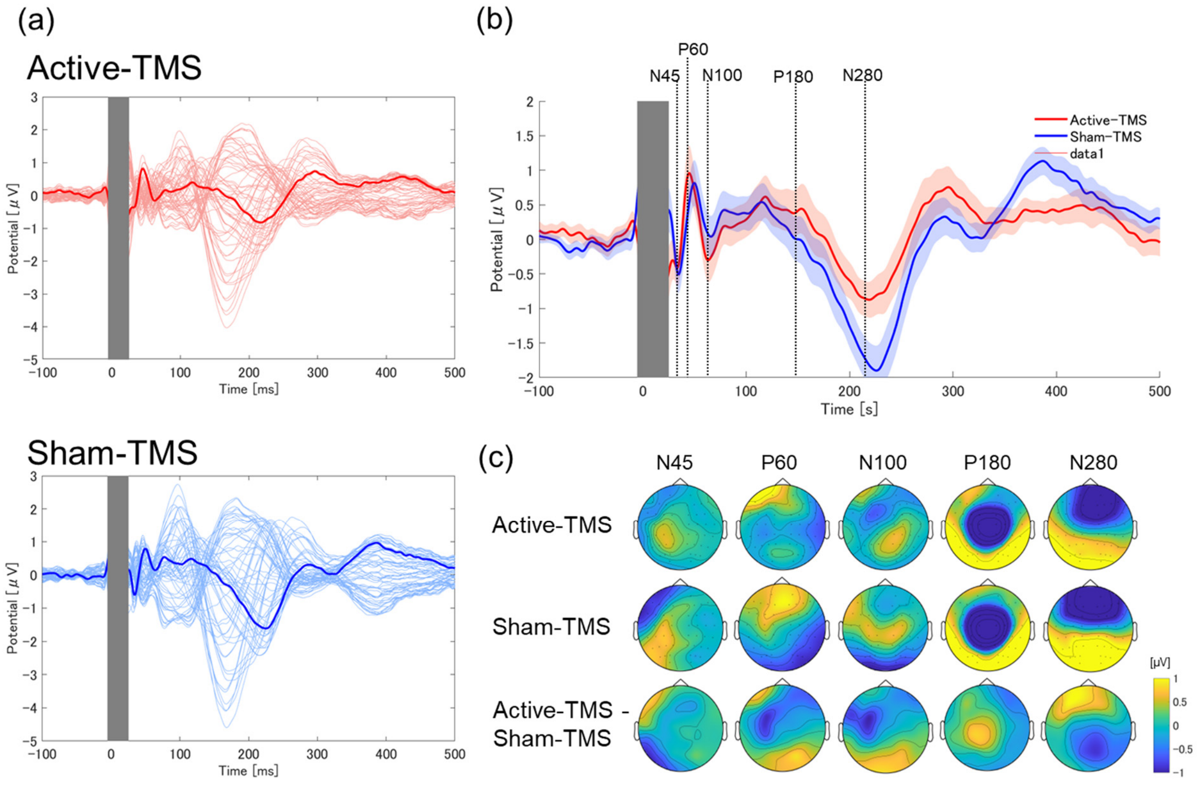

Figure S2. Butterfly plots for the active and sham conditions:

(a): The upper and lower panels show butterfly plots for the active and sham conditions, respectively. The TEP trace of each electrode is shown as a thin line and the average TEP of the DLPFC stimulation site electrodes as a thick line. EEG data from -5–30 ms immediately after TMS were excluded from the analysis because it is difficult to remove the effect of TMS artifacts, and the section is displayed as a gray bar. **(b):** The upper right panel shows the average TEP waveform for each stimulus condition shown on the left panels. The range of standard errors for each TEP waveform is indicated by red and blue shading, respectively. **(c):** The lower right panel shows the topoplots of the time window corresponding to each peak of the average TEP trace. The top, middle, and bottom topoplots indicate the active condition, the sham condition, and the active condition minus the sham condition, respectively.

Connectivity analysis with weighted phase lag index (wPLI):

Figure S3 shows the results of the active and sham conditions for the average wPLI of all participants in (a) θ-band (4-8 Hz), (b) α-band (8-13 Hz), (c) β-band (13-30 Hz), and (d) γ-band (30-100 Hz), respectively, and the difference between the two conditions The wPLI connectivity matrix for all electrodes (**Figure S3**) shows that in the θ and β bands, the active condition exhibits higher wPLI connectivity across the brain compared with the sham condition. In particular, focusing on the

stimulation sites (e.g., F5 electrode sites), the active condition showed enhanced wPLI connectivity in the β-band compared with the sham stimulation condition.

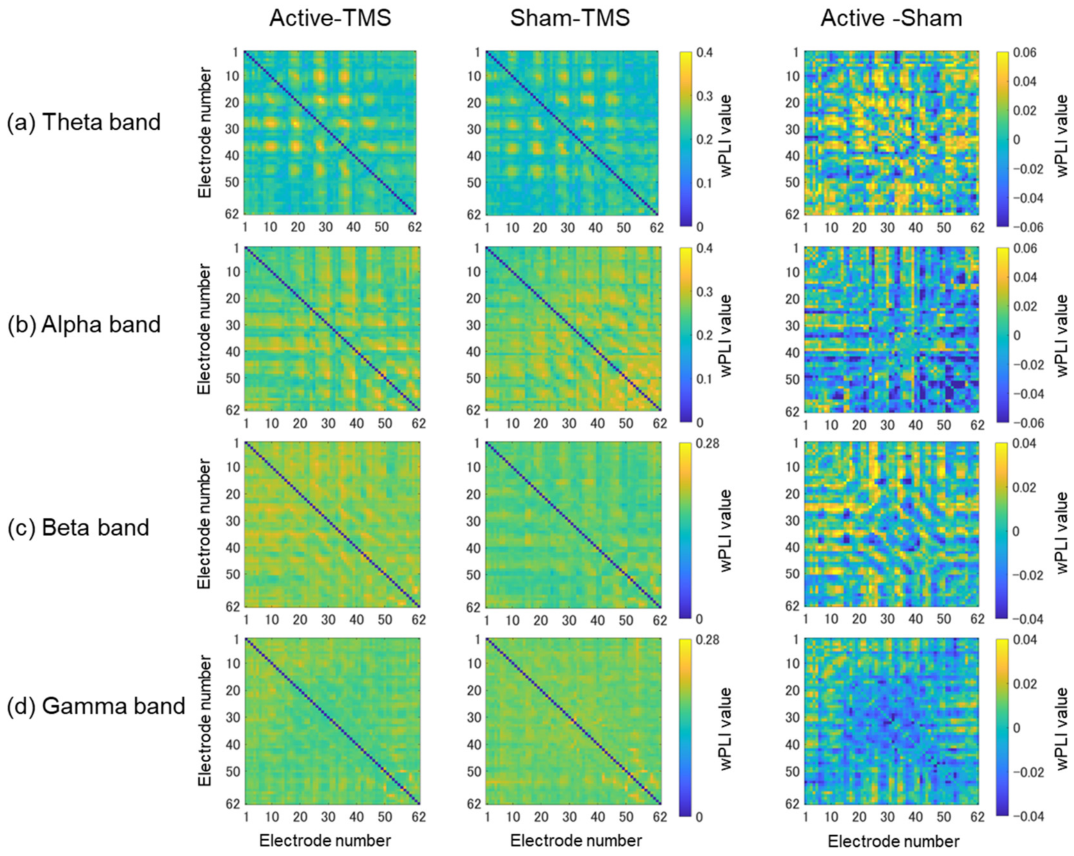

Figure S3. EEG connectivity matrices based on wPLI values in the active and sham conditions

Each panel shows the connectivity matrix (all electrode-pair connectivity maps) in (a) theta band (4–8 Hz), (b) alpha band (8–13 Hz), (c) beta band (13–30 Hz), and (d) gamma band (30–100 Hz), from top to bottom, respectively. On the other hand, the left side of the vertical column shows the wPLI values in the active condition, the center shows the wPLI values in the sham condition, and the right side shows the difference between the wPLI values in the active and sham conditions.

Table S1. The electrode sites identified as hubs by four graph-theory based indices: node degree (D), path length (PL), clustering coefficient (CC), and betweenness centrality (BC) in the active

			Active-TMS			
**Frequency band**	**Node**	**Node degree**	**Path length**	**Clustering Coefficient**	**Betweenness Centrality**	**Hub score**
θ	F5	54	0.15	0.19	0.03	2
	F8	50	0.16	0.19	0.03	2
	FT7	53	0.16	0.19	0.03	2
	FC5	52	0.15	0.18	0.02	2
	C5	53	0.16	0.19	0.02	2
	C1	55	0.17	0.22	0.005	2
	C2	56	0.18	0.23	0.001	2
	C6	51	0.15	0.19	0.02	2
	CP5	53	0.18	0.20	0.02	2
	CP1	56	0.18	0.23	0.002	2
	CP2	56	0.27	0.23	0.003	2
	CP6	49	0.22	0.24	0.001	2
	CP3	55	0.17	0.21	0.003	2
α	FT8	49	0.18	0.42	0.04	2
	C5	48	0.22	0.40	0.03	2
	C2	50	0.22	0.29	0.12	2
	T8	51	0.32	0.22	0.11	2
	CP5	48	0.22	0.19	0.08	2
	CP1	48	0.27	0.58	0.02	2
	CP2	43	0.22	0.11	0.11	2
	CP4	50	0.21	0.11	0.11	2
	P7	51	0.18	0.08	0.12	2
	P2	52	0.33	0.11	0.16	2
β	FC1	47	0.32	0.22	0.03	2
	C2	45	0.31	0.34	0.003	2
	CP3	47	0.21	0.36	0.002	2
	CP1	45	0.21	0.34	0.001	2
	CP2	45	0.21	0.42	0.009	2
	P3	49	0.22	0.33	0.01	2
	P1	48	0.30	0.44	0.005	2
	PZ	47	0.30	0.34	0.002	2
	P2	47	0.31	0.40	0.001	2
	P4	47	0.31	0.13	0.02	2

	FP1	49	0.32	0.42	0.02	2
	FP2	49	0.25	0.40	0.02	2
	AF3	49	0.35	0.29	0.03	2
	AF4	49	0.34	0.38	0.03	3
γ	F8	49	0.33	0.27	0.03	3
	C5	48	0.32	0.58	0.003	2
	TP7	44	0.32	0.60	0.002	2
	T8	46	0.33	0.34	0.04	3
	CP5	46	0.29	0.08	0.03	2

Sham-TMS						
Frequency band	Node	Node degree	Path length	Clustering Coefficient	Betweenness Centrality	Hub score
θ	F7	51	0.15	0.18	0.02	2
	F8	51	0.15	0.19	0.02	2
	C5	49	0.14	0.18	0.07	2
	C2	55	0.17	0.22	0.004	2
	C6	50	0.15	0.18	0.03	2
	CP5	51	0.15	0.19	0.03	2
	CP2	55	0.17	0.22	0.004	2
	CP4	55	0.16	0.21	0.005	2
	P1	55	0.17	0.21	0.005	2
	P2	55	0.17	0.21	0.004	2
α	CP1	49	0.18	0.23	0.10	2
	P1	49	0.19	0.22	0.07	2
	P2	46	0.22	0.13	0.07	2
	PO6	47	0.22	0.14	0.08	2
	PO8	49	0.24	0.14	0.01	2

	O2	47	0.21	0.11	0.07	2
	F6	48	0.33	0.11	0.05	2
β	F8	48	0.34	0.14	0.05	2
	FT8	47	0.33	0.17	0.04	2
	FP1	45	0.25	0.22	0.03	2
	AF3	45	0.23	0.22	0.03	2
	F8	46	0.22	0.36	0.04	3
	F6	47	0.24	0.33	0.02	3
γ	FC6	48	0.24	0.33	0.005	2
	FT8	47	0.23	0.36	0.03	3
	T7	48	0.28	0.57	0.008	2
	C6	47	0.21	0.56	0.008	2
	T8	46	0.22	0.59	0.01	3
	TP7	48	0.28	0.11	0.03	2
